# Novel Psychoactive Phenethylamines: Impact on Genetic Material

**DOI:** 10.3390/ijms21249616

**Published:** 2020-12-17

**Authors:** Veronica Cocchi, Sofia Gasperini, Patrizia Hrelia, Micaela Tirri, Matteo Marti, Monia Lenzi

**Affiliations:** 1 Department of Pharmacy and Biotechnology, Alma Mater Studiorum University of Bologna, 40126 Bologna, Italy; veronica.cocchi4@unibo.it (V.C.); sofia.gasperini4@unibo.it (S.G.); m.lenzi@unibo.it (M.L.); 2 Department of Translational Medicine, Section of Legal Medicine and LTTA Center, University of Ferrara, 44121 Ferrara, Italy; micaela.tirri@unife.it (M.T.); matteo.marti@unife.it (M.M.); 3 Collaborative Center for the Italian National Early Warning System, Department of Anti-Drug Policies, Presidency of the Council of Ministers, 44121 Ferrara, Italy

**Keywords:** phenethylamine, 2C-H, 2C-I, 2C-B, 25B-NBOMe, MDMA, genotoxicity, ROS, flow cytometry

## Abstract

Psychedelic and stimulating phenethylamines belong to the family of new psychoactive substances (NPS). The acute toxicity framework has begun to be investigated, while studies showing genotoxic potential are very limited or not available. Therefore, in order to fill this gap, the aim of the present work was to evaluate the genotoxicity by treating TK6 cells with 2C-H, 2C-I, 2C-B, 25B-NBOMe, and the popular 3,4-Methylenedioxymethylamphetamine (MDMA). On the basis of cytotoxicity and cytostasis results, we selected the concentrations (6.25–35 µM) to be used in genotoxicity analysis. We used the micronucleus (MN) as indicator of genetic damage and analyzed the MNi frequency fold increase by an automated flow cytometric protocol. All substances, except MDMA, resulted genotoxic; therefore, we evaluated reactive oxygen species (ROS) induction as a possible mechanism at the basis of the demonstrated genotoxicity. The obtained results showed a statistically significant increase in ROS levels for all genotoxic phenethylamines confirming this hypothesis. Our results highlight the importance of genotoxicity evaluation for a complete assessment of the risk associated also with NPS exposure. Indeed, the subjects who do not have hazardous behaviors or require hospitalization by using active but still “safe” doses could run into genotoxicity and in the well-known long-term effects associated.

## 1. Introduction

Psychoactive phenethylamines were among the first new psychoactive substances (NPS) to appear on the market in the late 1980s [1].

NPS are defined as “new narcotic or psychotropic drugs, in pure form or in preparation, that are not controlled by the United Nations drug conventions, but which may pose a public health threat comparable to that posed by substances listed in these conventions” [2]. The term “new” refers to substances which have recently become available on the illegal market which are not controlled by international drug laws and not necessarily developed by new inventions. In fact, various NPS are derived from pharmaceutical research patents which, due to their toxicological profile, have been eliminated from subsequent studies for the development of clinical trials [3]. NPS involve a large number of substances that are conventionally grouped into different classes according to their psychoactive effects. They include synthetic cannabinoids, stimulants, benzodiazepines, opioids, hallucinogens, and dissociatives [4,5].

To date, psychoactive phenethylamines represent the third largest group of NPS, after synthetic cannabinoids and cathinones, for a total of 99 molecules [6,7]. Also, some psychoactive phenethylamines were developed as potential drugs for psychiatric conditions [8,9,10], but they have not yet been put on the market due to their adverse effects or the lack of the desired therapeutic effects [11].

The chemist and pharmacologist Alexander Shulgin, considered the “godfather of psychedelics”, synthesized numerous phenethylamines and described them in his book, published in 1991, entitled “PiHKAL: A Chemical Love Story” where PiHKAL is the acronym for Phenethylamines I Have Known and Loved. From simple variations in the mescaline molecule, a hallucinogenic alkaloid mainly contained in peyote, other powerful psychedelic substances have been obtained, such as, for example, 4-bromo-2,5-dimethoxy phenethylamine (2C-B) synthesized by Shulgin in 1974 [7,12,13].

Psychoactive phenethylamines are a rather large group of molecules that includes both substances placed under the control of the 1961 and 1971 conventions and molecules that have recently appeared on the market and are not yet under the control of the United Nations conventions [14]. The first group includes, for example, amphetamines (α-methylphenylethylamine) and methamphetamines (N, α-dimethylphenylethylamine), among which we find MDMA, also known as ecstasy, one of the most popular drugs among young people but recently place side by side with other emerging molecules [15]. These molecules are divided into numerous sub-groups based on the different substitution on the aromatic ring, on the alkyl chain, and on the nitrogen atom. In particular, we can distinguish the “2C” series, characterized by methoxy groups in positions 2 and 5 and any other substituent on the aromatic ring (2C-B and 2C-I), the “D” series (DOI, DOC), similar to the 2Cs but with a methyl on the side chain, the “NBOMe” series, also made up of derivatives of the 2C series but with an N-benzyl-methoxy group (25B-NBOMe and 25C-NBOMe), the 4-Fluoroamphetamine (4-FA), “FLY” and “DragonFLY”, respectively tetrahydrobenzodifuranic (2C-B-Fly) and benzodifuranic (Bromo-DragonFLY) derivatives, and many others (for example p-methoxymethamphetamine or PMMA) [16].

These structural variations can produce different effects: many psychoactive phenethylamines exert a stimulating action, while some can act as entactogens, i.e., psychoactive substances that increase feelings of love and union with others, or that produce psychedelic effects [15,17,18].

In particular, these sought-after effects are due to the interaction with the dopaminergic, noradrenergic, and serotonergic systems. Phenethylamines can act both as stimulants by inhibiting the transporters for reuptake of norepinephrine (NET), dopamine (DAT), and serotonin (SERT), or the activity of vesicular monoamine transporter 2 (VMAT2), thus mimicking the effects of traditional drugs such as cocaine, amphetamine, methamphetamine and MDMA, and as hallucinogens by means of agonism on specific serotonin receptors, the 5-HT_2A,B,C_ [4,5,15,19,20,21].

Most of the studies currently available regarding the toxicity of psychoactive phenethylamines focus exclusively on the acute effects and mainly on the fatal and non-fatal acute intoxication cases due to psychedelics phenethylamines, in particular related to “NBOMe” [22,23,24,25] and “2C” [26,27,28] series intake. On the contrary, studies showing the potential long-term effects of psychoactive phenethylamines are very limited.

However, investigating this aspect is also of fundamental importance for a complete assessment of the risk associated with exposure to a substance. For example, a xenobiotic with mutagenic capacity could induce well-known long-term effects, including cardiovascular diseases [29,30,31] and numerous neuro-/chronic-degenerative diseases, such as Alzheimer’s [32,33,34] or cancer [35], in addition to the potential damage to the reproductive system, in terms of both impaired fertile capacity of the individual, induced malformations in his offspring, and the appearance of hereditary diseases [36,37].

Therefore, the NPS genotoxic potential must also be carefully investigated, as it is too often associated only with acute effects typically affecting the nervous system.

In the specific case of psychoactive phenethylamines, bibliographic research conducted on the main databases (i.e., Scopus from Elsevier and PubMed from MEDLINE), allowed us to identify only one publication on the in vivo genotoxicity of methamphetamine [38] and one of the cathinone mephedrone [39]. Moreover, currently, one study in vitro [40] and four studies in vivo [41,42,43,44] on DNA impact following the MDMA intake are reported, while, to our knowledge, there is no specific research regarding 2C-H, 2C-I, 2C-B and 25B-NBOMe.

In order to fill this large gap, we considered it necessary to plan the study presented in this work.

To analyze the possible genotoxicity of a molecule, numerous internationally validated in vitro tests are currently available, which are distinguished, for example, on the basis of the used test system (e.g., cells or bacteria) or on the basis of the type of the identified damage (e.g., gene mutations or chromosomal aberrations). In our laboratory, we have been using the MN test for some time due to its ability to highlight the genotoxic effects of both clastogenic and aneuploidogenic compounds. The MN is in fact a small nucleus generated by a fragment of a chromosome or an entire chromosome, and it is therefore used as a biomarker of chromosomal damage and genomic instability. OECD first adopted the in vivo version of this test, while in 2016 the current in vitro version corresponding to the guideline 487 was validated [45]. This guideline lists the possible cell lines suitable to analyze the presence of MNi, among which the most used are TK6 cells, by virtue of their human and non-tumoral origin, ease of maintenance in culture, and replicative speed.

**Figure 1:**
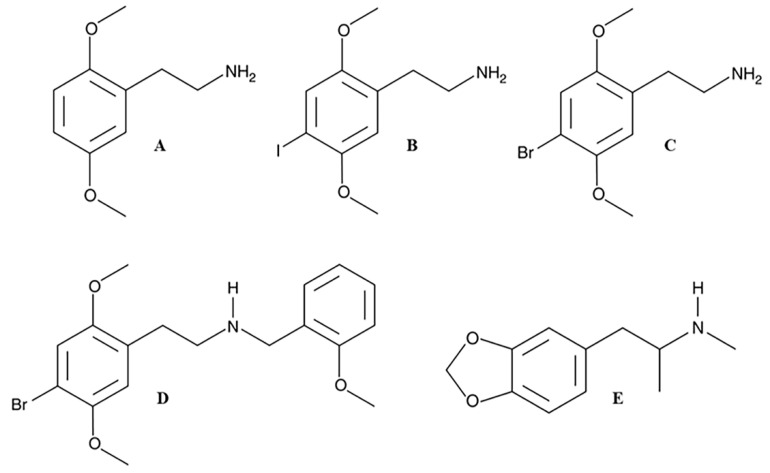
Chemical structures of 2C-H (**A**), 2C-I (**B**), 2C-B (**C**), 25B-NBOMe (**D**), and MDMA (**E**).

For these reasons, in the present work, we evaluated in human lymphoblastoid TK6 cells the genotoxic potential, in terms of increasing the frequency of MNi, of four psychedelic phenethylamines never tested, 2C-H, 2C-I, 2C-B and 25B-NBOMe, and included MDMA as an example of an entactogen/stimulant phenethylamine for which some genotoxicity data are already available in the literature (Figure 1).

In particular, in the first phase of the research, we selected the concentrations to be used in the subsequent genotoxicity analysis on the basis of the cytotoxicity and cytostasis induced by all chemical compounds under study, analyzed by flow cytometry through the Guava ViaCount assay and the Guava Nexin Assay. Subsequently, we used an automated flow cytometric protocol, recently developed in our laboratory, to analyze MNi frequency [46].

## 2. Results

In the preliminary phase of the research, we determined the concentrations to be used in the subsequent experiments aimed at evaluating the potential genotoxicity of different molecules under study. First, we assessed the 2C-H, 2C-I, 2C-B, 25B-NBOMe, and MDMA-induced cytotoxicity after 26 h treatment (time required by TK6 cells to carry out two cell cycles) by measuring the percentage of live cells at the different concentrations tested (6.25, 12.5, 25, 35, 50, 75 µM). This value was normalized on the one obtained in the untreated control cultures C (-) equal to 100% in order to check whether the cellular viability complied with the OECD threshold (equal to 55 ± 5%) [45].

**Figure 2:**
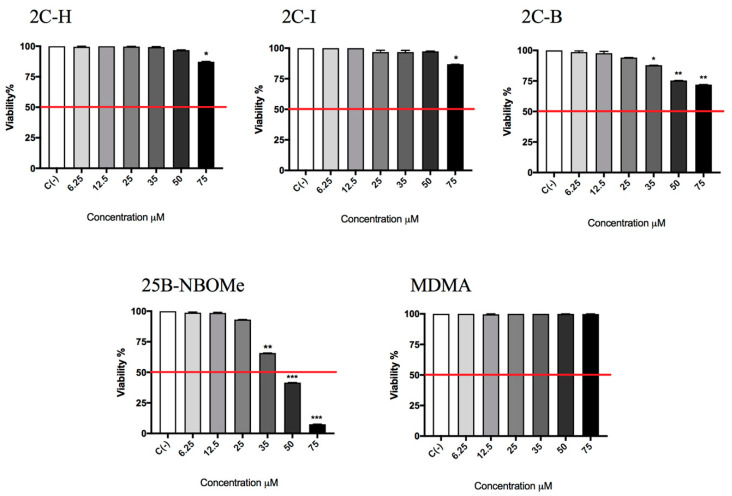
Cell viability on TK6 cells after 26 h treatment with 2C-H, 2C-I, 2C-B, 25B-NBOMe, and MDMA at the indicated concentrations with respect to the untreated control C (-). Each bar represents the mean ± SEM of five independent experiments. Data were analysed by ANOVA Repeated, followed by a Dunnet post-test. * *p* < 0.05 vs. C (-), ** *p* < 0.01 vs. C (-), *** *p* < 0.001 vs. C (-). The red line indicates the OECD threshold to be complied with for this genotoxicity test.

In Figure 2, it can be seen how the viability is well above the OECD threshold (represented by the red line) for all substances up to 75 µM, except for 25B-NBOMe (Figure 2).

In addition, to make the genotoxicity test reliable, it is necessary to check the cellular proliferation in order to verify that a sufficient number of cells has undergone mitosis and so transmitted the genetic damage suffered to the daughter cells. For this purpose, the OECD recommends the measurement of the Relative Population Doubling (RPD) to estimate the cytostasis and, analogously to the cytotoxicity, establishes a threshold at most equal to 55 ± 5% [45]. The concentrations that caused an RPD value well above 55 ± 5% were all the ones up to 35 µM for 2C-H, 2C-I and 2C-B, up to 12.5 µM for 25B-NBOMe and all the concentrations tested for MDMA (Table 1).

The research was continued by considering another alternative cell death mechanism, i.e., apoptosis, since the assessment of other cytotoxicity markers (e.g., cell integrity, apoptosis, necrosis…) could be useful for obtaining additional information, as the guideline n◦ 487 suggests in paragraph 27 [45].

**Figure 3:**
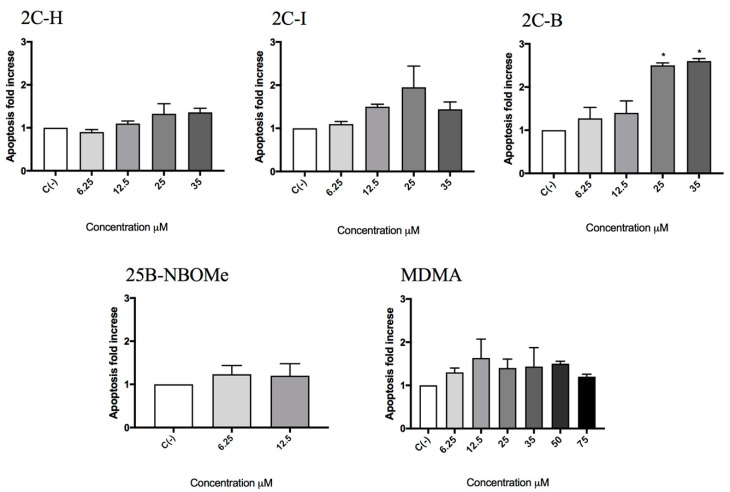
The apoptosis fold increase on TK6 cells after 26 h of treatment with 2C-H, 2C-I, 2C-B, 25B-NBOMe, and MDMA at the indicated concentrations with respect to the untreated control C (-). Each bar represents the mean ± SEM of five independent experiments. Data were analysed using repeated ANOVA followed by Bonferroni or Dunnet post-tests. * *p* < 0.05 versus C (-).

The evaluation of the apoptotic process was carried out according to the Guava Nexin assay. For 2C-H, 2C-I, 25B-NBOMe, and MDMA treated cultures, the induction of apoptosis never reached a doubling compared to the untreated control C (-), equal to 1, while for the 2C-B-treated culture a statistically significant increase of the apoptosis was observed at 25 µM and 35 µM (Figure 3).

Overall, the obtained results allowed us to select the concentrations to be used in the genotoxicity analysis. In particular, for 2C-H, 2C-I, and MDMA, 25 and 35 µM were tested and for 2C-B and 25B-NBOMe 6.25 and 12.5 µM.

These treated cultures were compared with untreated cultures (negative control) and Mytomicin (MMC) or Vinblastine (VINB) treated cultures (positive controls).

**Figure 4:**
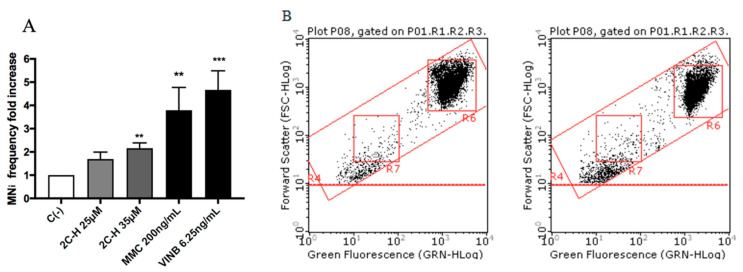
The MNi frequency fold increase on TK6 cells after 26 h of treatment with 2C-H at the indicated concentrations with respect to the untreated negative control C (-) and to positive controls [MMC and VINB]. (**A**) A plot of the nuclei and MNi in the untreated control (**B**, left), and in 35 µM 2C-H-treated cultures (**B**, right). Each bar represents the mean ± SEM of five independent experiments. Data were analysed using repeated ANOVA followed by the Bonferroni post-test. ** *p* < 0.01 vs. C (-); *** *p* < 0.001 vs. C (-).

**Figure 5:**
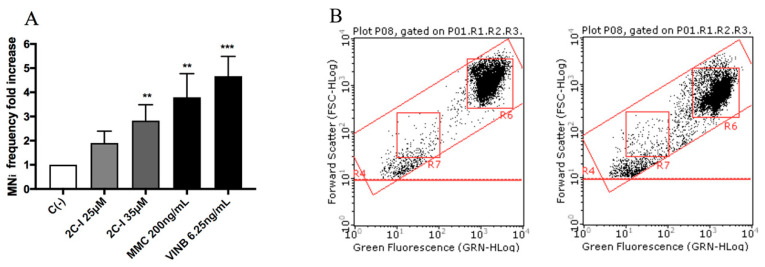
The MNi frequency fold increase on TK6 cells after 26 h of treatment with 2C-I at the indicated concentrations with respect to the untreated negative control C (-) and to positive controls [MMC and VINB]. (**A**) A plot of the nuclei and MNi in the untreated control (**B**, left), and in 35 µM 2C-I-treated cultures (**B**, right). Each bar represents the mean ± SEM of five independent experiments. Data were analysed using repeated ANOVA followed by Bonferroni post-test. ** *p* < 0.01 vs. C (-); *** *p* < 0.001 vs. C (-).

**Figure 6:**
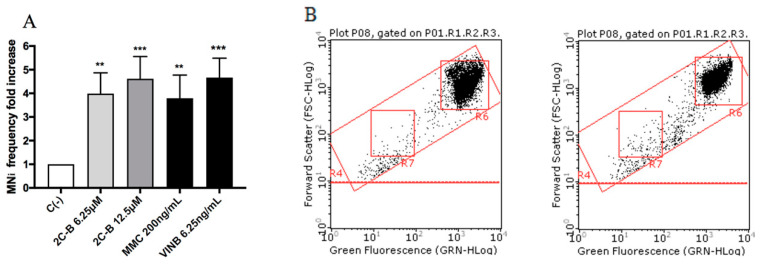
The MNi frequency fold increase on the TK6 cells after 26 h treatment with 2C-B at the indicated concentrations with respect to the untreated negative control C (-) and to positive controls [MMC and VINB]. (**A**) A plot of the nuclei and MNi in the untreated control (**B**, left), and in 12.5 µM 2C-B-treated cultures (**B**, right). Each bar represents the mean ± SEM of five independent experiments. Data were analysed using repeated ANOVA followed by the Bonferroni post-test. ** *p* < 0.01 vs. C (-); *** *p* < 0.001 vs. C (-).

**Figure 7:**
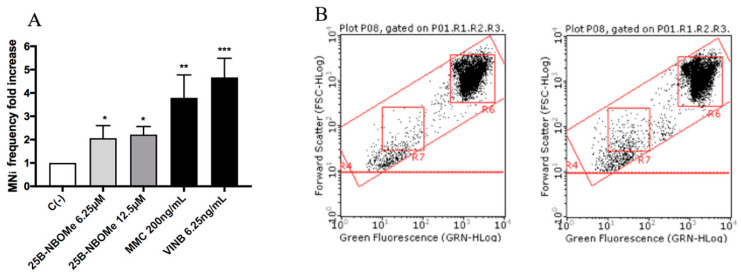
The MNi frequency fold increase on TK6 cells after 26 h treatment with 25B-NBOMe at the indicated concentrations with respect to the untreated negative control [C (-)] and to positive controls [MMC and VINB]. (**A**) The plot of the nuclei and MNi in the untreated control (**B**), and in 12.5 µM 25B-NBOMe -treated cultures (**C**). Each bar represents the mean ± SEM of five independent experiments. Data were analysed using repeated ANOVA followed by Bonferroni post-test. * *p* < 0.05 vs. C (-), ** *p* < 0.01 vs. C (-); *** *p* < 0.001 vs. C (-).

**Figure 8:**
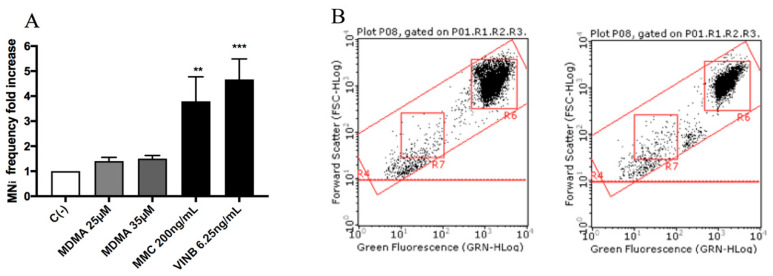
MNi frequency fold increase on TK6 cells after 26 h treatment with MDMA at the indicated concentrations with respect to the untreated negative control C (-) and to positive controls [MMC and VINB]. (**A**) A plot of the nuclei and MNi in the untreated control (**B**, left) and in 35 µM MDMA-treated cultures (**B**, right). Each bar represents the mean ± SEM of five independent experiments. Data were analysed using repeated ANOVA followed by Bonferroni post-test. ** *p* < 0.01 vs. C (-); *** *p* < 0.001 vs. C (-).

All molecules under study were genotoxic (Figure 4, Figure 5, Figure 6 and Figure 7) except for MDMA (Figure 8), as demonstrated by the MNi frequency fold increase. In particular, 2C-H and 2C-I showed a statistically significant increase of the MNi frequency at the 35 µM concentration (Figure 4 and Figure 5), while 2C-B and 25B-NBOMe increased at both the tested concentrations, 6.25 µM and 12.5 µM (Figure 6 and Figure 7).

Lastly, in order to identify a possible mechanism of action at the basis of the genotoxic activity, TK6 cells were treated for 1 h with the highest concentration tested for each substance: 35 µM for 2C-H, 2C-I and MDMA, and 12.5 µM for 2C-B and 25B-NBOMe, and then the possible ROS induction was measured.

**Figure 9:**
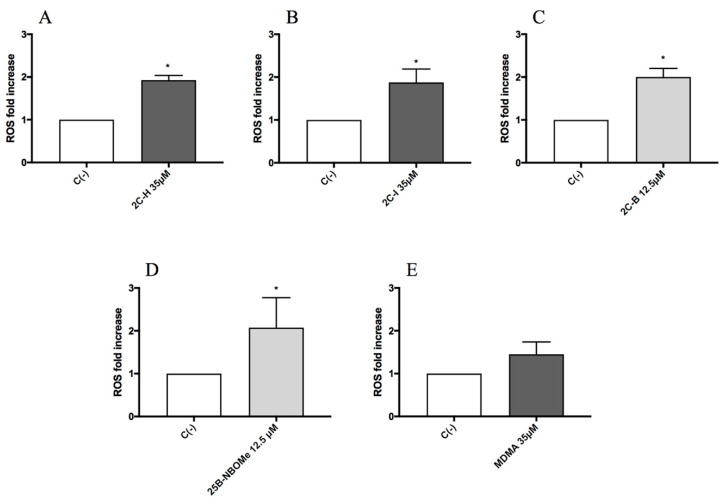
The ROS fold increase on TK6 cells after 1 h of treatment with 2C-H (**A**), 2C-I (**B**), 2C-B (**C**), 25B-NBOMe (**D**), and MDMA (**E**) at the indicated concentrations with respect to the untreated negative control C (-). Each bar represents the mean ± SEM of five independent experiments. Data were analysed using repeated ANOVA followed by *t*-test for paired data. * *p* < 0.05 vs. C (-).

The results showed a statistically significant increase of the mean fluorescence intensity with respect to the untreated negative control C (-), accounted equal to 1, of about two-fold increase, for all the compounds except for MDMA (Figure 9A–E).

## 3. Discussion

Our results allow us to highlight, for the first time, the genotoxic capacity of the four psychedelic phenethylamines under study, 2C-H, 2C-I, 2C-B, and 25B-NBOMe, at the selected sub-cytotoxic and sub-cytostatic concentrations.

In fact, the Guava ViaCount assay showed that 2C-H, 2C-I, and 2C-B did not induce cytotoxicity and cytostasis up to 35 µM, 25B-NBOMe up to 12.5 µM.

The cytotoxicity test used did not render the apoptotic cells distinguishable, but the apoptosis induction can be a consequence of cellular genetic damage that the cell was unable to repair. Therefore, we found it necessary to proceed with a more specific test to highlight this alternative death mechanism [46,47]. The results obtained with the Guava Nexin assay showed similar behavior for all the substances analyzed except for 2C-B. In fact, 2C-B stimulated the apoptosis of TK6 cells only at the highest concentrations tested (25 and 35 µM). This fact is of crucial toxicological importance and has great repercussions in terms of genotoxicity, because it underlines the inability of the cell to counteract, through this selective death mechanism, the transmission of the genetic damage suffered from the mother cell to daughter cells.

Overall, on the basis of the obtained results, the concentrations to be used for the evaluation of genotoxicity were selected, and we proceeded with the MN test. After carrying out the treatments, we waited for 26 h before processing the samples to allow the TK6 cells to carry out two replicative cycles, thus fixing the possible genetic damage and transmitting it to the progeny.

At the end of the treatment time, to analyze the MNi frequency in treated and untreated cultures, we used a new automated flow cytometric protocol that we recently developed in our laboratory and published. This protocol has numerous advantages over the standard procedure via optical microscopy, including speed and economic efficiency, as it reduces sample preparation and analysis times by up to 80% with considerable cost savings. The automation of the analysis also makes it possible to overcome the problem of the subjectivity of the interpretation by the operator and to analyze a ten-fold greater number of events [46]. In view of these undeniable advantages, we used this protocol in our previous publication regarding the genotoxicity of some synthetic cannabinoids, confirming its usefulness, validity, and efficacy. In fact, it allowed us to analyze 6 different compounds in a short time, highlighting their genotoxic properties even at low concentrations with more objective and statistically robust results [47].

The natural next step was therefore to use the same protocol to analyze the genotoxicity of molecules belonging to another group of NPS, such as psychoactive phenethylamines. In the present work, our automated cytofluorimetric protocol was able to demonstrate a statistically significant increase in MNi frequency for all four psychedelics phenethylamines, in particular for 2C-H and 2C-I at the highest concentration tested (35 µM), and for 2C-B and 25B-NBOMe at both tested concentrations (6.25 and 12.5 µM).

From a structural point of view, Figure 1 showed common chemical characteristics of the 2C-X series (A-B-C) responsible for its psychedelic properties: the two methoxylic groups (respectively in position 2 and 5) and a lipophilic substituent (in position 4) on the phenethylamine ring. Moreover, for 25B-NBOMe (Figure 1D; derived from the 2C-B structure) the addition of the substituent N-(2-methoxy)benzyl greatly improves its potency compared to both its predecessors 2C-B and MDMA [19,24].

The obtained results confirm what has been reported in the literature with regard to the correlation between chemical structure and different genotoxic potency. In fact, it seems that the molecules that have a halogen close to a double bond show greater genotoxic capacity [48] and that, in particular, the brominated compounds are more powerful than those containing a different halogen [49]. Indeed, even in our case, 2C-B and 25B-NBOMe were found to be stronger genotoxic agents than 2C-I and 2C-H. 25X-NBOMe compounds are ultrapotent and highly efficacious agonists of serotonin 5-HT_2A_ and 5-HT_2C_ receptors (Ki values in low nanomolar range), with more than 1000-fold selectivity for 5-HT_2A_ compared with 5-HT_1A_, and they have higher selectivity and affinity for the serotonin 5-HT_2A_ receptor than their corresponding compounds of the 2C-X series [7,50,51]. This pharmacokinetic profile is reflected in their more potent in vitro activity and psychedelic and even toxic in vivo effect [23,52,53,54]. In vitro studies have shown that 25X-NBOMe have greater potency on 5HT_2A_ receptors (EC50 = 0.758–0.819 nM) than 2C-X compounds (EC50 = 3.16–8.46 nM) [55], and this agrees with the in vivo studies showing that 25X-NBOMe induced a head twitch response in rodents (HTR; a behavioral marker in rodents for hallucinogen effects in humans) with a potency several-fold higher than their 2C-X counterparts [56,57,58]. These preclinical studies are in agreement with the dosages used by consumers. In fact, the active dosages of 25X-NBOMe range from 20 to 100 times lower than those of the compounds of the class 2C-X [23,59,60,61,62].

MDMA did not show a cytotoxic and cytostatic effect at all concentrations tested and did not increase the MNi frequency in any case.

These results agree with those obtained by Yoshioka et al. on a hamster pulmonary fibroblasts cell line (CHL/IU), where the authors demonstrated, by the MN test and by the chromosomal aberration test, the genotoxicity of N-nitroso-3,4-methylenedioxymethamphetamine (N-MDMA), but not of the MDMA as such. In fact, MDMA is a secondary amine able to react in acidic environments, such as the stomach, with N-nitroso compounds, often present in many foods and other sources, to form N-nitrosamines, which are known carcinogen compounds [40].

The results of this study and those obtained in our work confirm the well-known key role of metabolism in chemical genotoxicity [63]. Indeed, the apparent lack of the genotoxic capacity of MDMA is probably due to the poor metabolic capacity of the cell line used in this study. In fact, TK6 cells are recognized as an assay system validated by the OECD for in vitro genotoxicity test, but it has been demonstrated that human TK6 cells have negligible expression of the major Cytocromes P450 (CYPs) responsible for metabolic transformation of chemicals [64]. For example, the well-characterized genotoxicant, benzo [a] pyrene (B [a] P) failed to induce micronuclei in TK6 cells because CYP1A1, 1B1, and 1A2 are not expressed [65].

The in vitro genotoxicity test has been and will continue to be recommended for identifying DNA reactive chemicals for hazard identification and the in vitro MN test is one of the short-term assays supported by international authorities, such as the International Council for Harmonization, (ICH) for in vitro genotoxicity assessment [66]. The mouse lymphoma L5178Y cells and human lymphoblastoid TK6 cells are the most commonly used cell lines for genotoxicity testing [67]. To overcome the lack of capabilities, the international guidelines suggest a source of exogenous metabolic activation in genotoxicity analyses, such as the S9 mix. In this paper, we did not consider it necessary to investigate the effects of metabolites by adding the S9 mix to the cell cultures because, except for MDMA, all the other four phenethylamines under study are genotoxic by themselves, i.e., the parental compound is already able to increase the frequency of MN compared to the control cultures in a statistically significant way, even if not metabolically activated.

Not having planned metabolite genotoxicity studies certainly represents a limitation in our work which future studies will have to overcome.

The study ended by analyzing the phenethylamines ROS induction in order to identify a possible mechanism underlying the genotoxic activity demonstrated. In fact, it has long been known how ROS such as ^1^O_2,_ O_2_^•−^, H_2_O_2_, ^•^OH, are involved in genetic damage [68,69,70]. Our results highlighted a statistically significant increase in ROS levels for all genotoxic phenethylamines.

These findings allow us to hypothesize a possible involvement of oxidative stress in the DNA damage induced by 2CH, 2C-I and 25B-NBOMe. Moreover, it is important to underline that the ROS assay did not highlight any ROS level increase induced by MDMA. These results agree with the fact that MDMA was not genotoxic in our study and with what has been demonstrated by Valente et al. Indeed, researchers tested MDMA in undifferentiated and differentiated human SH-SY5Y cells demonstrating, also in this case, that MDMA was unable to trigger the production of ROS [70]. On the contrary, the same author in another paper highlighted an MDMA-induced increase of ROS levels at 24 h, but in a cell line with a different metabolic capacity compared to the TK6 cells used in our study, i.e., primary rat hepatocytes [71]. Moreover, Zhou et al. demonstrated in C2C12 myoblast cells an increase in the mitochondrial superoxide production, following MDMA exposure, but at concentrations 30 times higher than those used in our study and for a much longer treatment time (24 h vs. 1 h) [72]. According to our knowledge, no studies are available regarding the possible oxidative stress induced by 2C-H, 2C-I, 2C-B, and 25B-NBOMe, but there are data regarding related compounds such as cathinones MDPV, αPVP, and methylone, which demonstrate their ability to cause an increase in superoxide levels of SH-SY5Y after 2–24 h treatment in a concentration-dependent manner (1000–3000 μM) [70,72,73]. Moreover, in another paper MDPV, mephedrone and naphyrone increased O_2_^•−^ levels in HepG2 after 24 h treatment at concentrations of 2 mM, 1 mM, and 0.2 mM, respectively [74]. Metaphedrone mediates oxidative stress at 10 μM after 24 h in the primary rat hepatocyte of a variety of cell models [75]. With regards to amphetamines, Luethi et al. investigated oxidative stress in HepG2 cells, highlighting, for example, the ability of amphetamine, 4-fluoroamphetamine, and 4-chloroamphetamine to increase mitochondrial superoxide levels [76].

Therefore, our results are, in general, in accordance with what is currently reported in the literature, and they are even more impactful in light of the fact that in our case the induction of ROS is already visible after only 1 h of treatment and at such low concentrations that they do not minimize cell viability, neither after 1 h nor 24 h of exposure.

A final consideration must be made with regard to the concentrations tested. It could be asked whether they are reachable in vivo and comparable to the level of phenethylamine detectable in the human serum upon intake. However, it is recognized that for genotoxic substances, zero risk corresponds only to zero doses, and therefore, any dose is potentially toxic. Indeed, it is not possible to define a No-Observed-Adverse-Effect Level (NOAEL) for genotoxic substances, but by simply increasing the dose and the exposure the likelihood that damage will occur increases [77].

In conclusion, the present work showed us for the first time the impact of 2C-H, 2C-I, 2C-B, and 25B-NBOMe phenethylamines on genetic material and allows us to hypothesize a key role of oxidative stress in the induced DNA damage.

This evidence is very important from a toxicological and forensic point of view since, up to now, only acute poisonings following intake of medium-high doses of psychoactive phenethylamines have been observed in medical centers and described in clinical reports. The hypothesis that the repeated consumption of 2C-H, 2C-I, 2C-B, and 25B-NBOMe at low doses could proceed without people reporting serious acute side effects could appear reassuring, but instead is potentially alarming. Indeed, this phenomenon would be difficult or even impossible to detect if we analyzed only subjects who have hazardous behaviors or require hospitalization. In fact, there would be a potential “grey zone” of subjects whose use or abuse would not be screened [78]; these subjects, by using active but still “safe doses” of 2C-H, 2C-I, 2C-B and 25B-NBOMe, could run into mutagenesis and carcinogenesis processes.

## 4. Materials and Methods

### 4.1. Reagents

Ethylenediaminetetraacetic acid (EDTA), fetal bovine serum (FBS), L-glutamine (L-GLU), MMC, Nonidet, penicillin-streptomycin solution (PS), potassium chloride, potassium dihydrogen phosphate, Roswell Park Memorial Institute (RPMI) 1640 medium, water bpc grade, ethanol, sodium chloride, sodium hydrogen phosphate, VINB, 2′-7′-dichlorodihydrofluorescin diacetate (DCFH-DA) (all purchased from Sigma-Aldrich, St Louis, MO, USA), Guava Nexin Reagent, Guava ViaCount Reagent (all purchased from Luminex Corporation, Austin, TX, USA), RNase A, SYTOX Green, 7-aminoactinomycin D (7-AAD) (purchased from Thermo Fisher Scientific, Waltham, MA, USA).

### 4.2. Synthetic Phenethylamines

The phenethylamines 2C-H, 2C-I, 2C-B, 25B-NBOMe, MDMA were purchased from LGC Standards (LGC Standards S.r.L., Sesto San Giovanni, Milan, Italy) and www.chemicalservices.net.

All synthetic phenethylamines were dissolved in absolute ethanol up to 10 mM stock solution and stored at −20 °C. The absolute ethanol concentration was always in the range of 0.25–0.75% in all experimental conditions to avoid potential solvent toxicity.

### 4.3. Cell Culture and Treatments

Human TK6 lymphoblast cells were purchased by Sigma-Aldrich (St. Louis, Missouri, USA) and were grown at 37 °C and 5% CO_2_ in RPMI-1640 supplemented with 10% FBS, 1% L-GLU, and 1% PS. To maintain exponential growth and considering that the time required to complete the cell cycle is 13–14 h, the cultures were divided every three days in fresh medium, and the cell density did not exceed the critical value of 9 × 10^5^ cells/mL.

In all experiments, aliquots of 2 × 10^5^ of TK6 cells were treated with increasing concentrations of phenethylamines from 0 to 75 µM and incubated for 26 h, corresponding to two replication cycles for the analysis of cytotoxicity, cytostasis, apoptosis, and genotoxicity, while for the analysis of ROS, 1 h of treatment was performed.

### 4.4. Flow Cytometry

All flow cytometry analysis reported below were performed using a Guava easyCyte 5HT flow cytometer equipped with a class IIIb laser operating at 488 nm (Luminex Corporation, Austin, TX, USA).

#### 4.4.1. Cytotoxicity Analysis

Cytotoxicity assay was performed as previously described by Lenzi et al. [46,47]. Briefly, at the end of the phenethylamines treatment time, 1.5 × 10^5^ cells were stained with Guava ViaCount Reagent. The reagent contains the Propidium Iodide (PI) that allows us to discriminate viable from dead cells based on different permeability to DNA-binding dye.

180 μL of reagent were added to 20 μL of cell suspension and incubated for 5 min at room temperature (RT). After, a total of 1000 events were acquired and analyzed by Guava ViaCount software.

The results obtained from the samples treated with different concentrations of phenethylamines were normalized on those obtained from the untreated control cultures, accounted equal to 100%. These results were used to check that the cellular viability percentage respected the OECD threshold (55 ± 5%) for each phenethylamine treatment [45].

#### 4.4.2. Cytostasis Analysis

In parallel, always using the Guava ViaCount Reagent, the number of cells seeded at time 0 and that measured at the end of the phenethylamines treatment time was used to check the correct replication in the control cultures and to compare it to that measured in the treated cultures by the RPD, calculated as the following formula [46,47]:

$$\mathrm{RPD}=\;\frac{\left(No.\;\;of\;Population\;doublings\;in\;treated\;cultures\right)}{\left(No.\;\;of\;Population\;doublings\;in\;control\;cultures\right)}\;\times \;100$$

Similarly to cytotoxicity, the cytostasis was checked in order to verify that cell proliferation respected the threshold established by the OECD guideline (55 ± 5%) [45].

#### 4.4.3. Apoptosis Analysis

The percentage of apoptotic cells was evaluated by the Guava Nexin Assay according to the procedure by Lenzi et al. [46,47].

Briefly, at the end of the phenethylamines treatment time, the percentage of apoptotic cells was assessed using the Guava Nexin Reagent. The reagent contains the 7-aminoactinomycin D (7-AAD), a cell non-permeant dye, that allows discrimination between live and dead cells and the Annexin-V-PE that allows the identification of apoptotic cells by binding the phosphatidylserine on the cell surface.

100 μL of reagent were added to 100 μL of cell suspension (~1 × 10^5^ cells) and incubated for 20 min at RT. After, a total of 2000 events were acquired and analyzed by Guava Nexin software.

The apoptotic cell percentages recorded in the cultures treated with phenethylamines different concentrations were normalized on those recorded in the untreated control cultures, accounted equal to 1 and expressed as apoptotic fold increase. These results were used to check that the apoptosis induction for each phenethylamine was similar or corresponding at most to a doubling of that recorded in the untreated control cultures.

#### 4.4.4. Genotoxicity Analysis

The analysis of the MNi frequency was performed using a new automated flow cytometric protocol that we recently developed in our laboratory. The detailed protocol is described in the paper by Lenzi et al. [46] and was used in our previous publication to analyze the genotoxicity of some synthetic cannabinoids [47].

Briefly, at the end of the phenethylamines treatment time, 7 × 10^5^ cells were collected and incubated with 7-AAD at RT for 5 min, after which the cells were lysed and stained with SYTOX Green for 1 h. After, a total of 10,000 nuclei, derived from viable and proliferating cells (7-AAD negative), and the corresponding number of micronuclei were acquired. The discrimination between nuclei and MNi occurs on the basis of the different size analyzed by Forward Scatter (FSC), and the different intensity of green fluorescence (SYTOX Green).

The MNi frequencies (number of MNi/10,000 nuclei) recorded in treated cultures were normalized on those recorded in the untreated control cultures, accounted equal to 1 and expressed as MNi frequency fold increase.

As recommended by OECD guideline 487, we used MMC and VINB as positive controls, being known clastogen and aneuploidogen agents, respectively [45].

#### 4.4.5. ROS Analysis

At the end of the phenethylamines treatment time, 2 × 10^5^ cells were centrifuged, resuspended in PBS1X to measure intracellular ROS level and stained at 37 °C in the dark for 20 min with 2′,7′-dichlorodihydrofluorescin diacetate (DCFH2-DA). After, a total of 5000 events, derived from viable cells, were acquired and analyzed using Guava Incyte software.

The fluorescence intensity of 2′,7′-dichlorofluorescin (DCF) (which forms in cells in the presence of ROS) recorded in treated cultures was normalized on those recorded in the untreated control cultures, accounted equal to 1 and expressed as a ROS fold increase.

### 4.5. Statistical Analysis

All results were expressed as the mean ± SEM of at least five independent experiments. For the statistical analysis of viability, apoptosis, and MNi, we used the Analysis of Variance for paired data (repeated ANOVA), followed by Dunnett or Bonferroni tests as the post-test. For the statistical analysis of ROS intracellular levels, we used the t-test for paired data. All the statistical analyses were performed using Prism Software 4.

## Figures and Tables

**Table 1:** RPD on TK6 cells after 26 h treatment with 2C-H, 2C-I, 2C-B, 25B-NBOMe and MDMA at the indicated concentrations with respect to the untreated control C (-). Data are presented as the mean ± the SEM of five independent experiments.

Relative Population Doubling (RPD)					
	2C-H	2C-I	2C-B	25B-NBOMe	MDMA
**C (-)**	100.00%	100.00%	100.00%	100.00%	100.00%
**6.25 µM**	97.5% ± 0.9	90.9% ± 0.7	95.9% ± 0.7	86.4% ± 1.3	96.0% ± 1.0
**12.5 µM**	81.1% ± 1.8	89.6% ± 2.2	93.1% ± 2.1	81.2% ± 2.0	96.0% ± 0.8
**25 µM**	85.0% ± 2.4	72.5% ± 2.0	69.7% ± 1.6	50.0% ± 0.8	98.7% ± 0.9
**35 µM**	74.0% ± 1.8	63.9% ± 1.6	63.1% ± 1.6	1.0% ± 0.2	98.0% ± 1.5
**50 µM**	34.1% ± 1.2	54.8% ± 0.9	10.0% ± 1.8	0.0	96.0% ± 1.2
**75 µM**	13.0% ± 1.5	30.8% ± 1.0	0.0	0.0	91.9% ± 1.3

## Abbreviations

2C-B	2,5-dimethoxy-4-bromophenethylamine
2C-H	2,5-dimethoxyphenethylamine
2C-I	2,5-Dimethoxy-4-iodophenethylamine
25B-NBOMe	4-bromo-2,5-dimethoxy-*N*-[(2-methoxyphenyl)methyl]-benzeneethanamine
DCF	2′,7′-dichlorofluorescin
DCFH-DA	2′,7′– dichlorofluorescin diacetate
DCFH2-DA	2′,7′-dichlorodihydrofluorescin diacetate
DAT	Dopamine Transporter
FCS	Forward Scatter
MDMA	3,4-methylenedioxy-methamphetamine
MMC	Mitomycin
MN	Micronucleus
NET	Norepinephrine Transporter
NPS	New Psychoactive Substances
OECD	Organization of Economic Cooperation and Development
PI	Propidium Iodide
ROS	Reactive Oxygen Species
RPD	Relative Population Doubling
SERT	Serotonin Transporter
VINB	Vinblastine
